# Risk factors associated with peritoneal carcinomatosis of gastric cancer in staging laparoscopy: A systematic review and meta-analysis

**DOI:** 10.3389/fonc.2022.955181

**Published:** 2022-10-28

**Authors:** Guangmin Guan, Zhemin Li, Qi Wang, Xiangji Ying, Fei Shan, Ziyu Li

**Affiliations:** Key Laboratory of Carcinogenesis and Translational Research (Ministry of Education/Beijing), Gastrointestinal Cancer Center, Peking University Cancer Hospital and Institute, Beijing, China

**Keywords:** gastric cancer, peritoneal carcinomatosis, risk factors, staging laparoscopy, indications

## Abstract

**Background:**

The optimal indications of staging laparoscopy in gastric cancer to detect peritoneal carcinomatosis are still controversial. We performed this systematic review and meta-analysis to quantify the relevance of the preoperative factors with peritoneal carcinomatosis to explore the indications of staging laparoscopy.

**Materials and methods:**

Systematic searches were conducted using Medline, Embase, and the Cochrane Library in December 2021. On the basis of calculating the odds ratio (OR) of each factor, we quantified the association between the risk factors and peritoneal carcinomatosis such as clinical T/N stage, Borrmann type, and tumor markers, using meta-analysis with a random-effects model.

**Results:**

A total of 21 case-control studies and one cohort study were obtained. T stage, N stage, and differentiation degree were most widely studied, with OR values of 2.96 (95% CI: 1.87–4.69), 1.22 (95% CI: 0.86–1.73), and 1.91 (95% CI: 1.42–2.56), respectively. Among all the factors, elevated CA125 (OR = 19.45, 95% CI: 4.71–80.30), Borrmann type IV (OR = 7.68, 95% CI: 3.62–16.27), and large tumor diameter (OR = 5.12, 95% CI: 2.55–10.31) had the highest OR. In particular, CA125 had the best predictability for peritoneal carcinomatosis but was only mentioned by three articles.

**Conclusions:**

There was a cognitive gap between the awareness and importance of risk factors for peritoneal carcinomatosis. In addition to T4 stage, patients with factors with high OR, such as Borrmann type IV, large tumor diameter, and elevated CA125, should undergo staging laparoscopy.

## Introduction

There were nearly 1.07 million new cases of gastric cancer in 2020, with incidence ranking fifth and mortality ranking fourth among malignant tumors (1). Peritoneal carcinomatosis (PC), including macroscopic carcinomatosis (P1) and positive cytology (CY1), is the most common metastasis of gastric cancer (2, 3). Current examination methods, such as CT and MRI, are not effective in detecting PC. It was reported that the sensitivity of CT in diagnosing PC changed from 25% to 50.9% and often at a relatively advanced stage (4–6). There is no evidence that PC could be diagnosed effectively by MRI.

The guidelines for gastric cancer recommend staging laparoscopy (SL) combined with peritoneal cytology as the best method for detecting PC (7–9). Its sensitivity changes from 85% to 98% and its specificity is close to 100% (10, 11). However, SL needs to be carried out under general anesthesia, which will increase the cost to patients, and SL could be more cost-effective if used selectively (12). However, the indications for SL of the guidelines are inconsistent. Japanese institutions chose patients with a more advanced stage like bulky lymph nodes or large Borrmann type III to undergo SL (7), whereas Western countries tend to apply SL to all patients with resectable tumors (13). This study aims to summarize the preoperative risk factors of PC to screen patients that are suitable for SL.

## Materials and methods

### Literature search strategy

The study was performed following the PRISMA statements for systematic reviews and meta-analyses including observational studies. We searched the databases of PubMed, Embase, MEDLINE, and the Cochrane Library for studies that were published before December 2021 using the search strategy “((gastric [Title/Abstract]) OR (stomach [Title/Abstract]) OR (gastroesophageal [Title/Abstract])) AND ((staging laparoscopy [Title/Abstract]) OR (diagnostic laparoscopy [Title/Abstract]))”. In addition, the references cited in the publications were manually searched to identify additional relevant studies. Only studies published in English were included. No institutional review board approval was required for this literature review. This review was not registered.

### Inclusion and exclusion criteria

Studies included in this systematic review and meta-analysis were required to meet the following criteria: 1. patients were pathologically diagnosed with gastric cancer; 2. PC was diagnosed by SL or open surgery; and 3. PC was diagnosed before any anti-tumor treatment. Studies were excluded on the basis of the following criteria: 1. patients were diagnosed with recurrent gastric cancer; 2. patients were diagnosed clearly as stage IV or with distant metastasis by CT or other non-invasive methods; 3. patients suffered from other malignant tumors at the same time; and 4. patients had a history of malignant tumors.

### Data extraction and quality assessment

Two investigators (GMG and ZML) independently screened titles, abstracts, and full texts of the studies for eligibility. Disagreement was resolved between the two reviewers through discussion or, if needed, adjudication by a third reviewer (ZYL). The following information from the included studies were collected in the same way: first author, year of publication, country of patients, duration of study, inclusion and exclusion criteria, sample size, median or mean age, gender, the definition of PC, diagnostic methods of PC, and OR values of risk factors. We would extract the original data to calculate it when feasible, if needed. More specifically, in the latest Japanese guideline for gastric cancer, both P1 and CY1 were defined as M1 stage, so we did the same (7).

### Statistical analysis

We quantified the association between risk factors and PC such as age and gender, using meta-analysis with a random-effects model, and presented the results with forest plots. Heterogeneity was tested with Cochran’s Q-test, with P-value < 0.1 indicating heterogeneity, and quantified by the I² statistic with values of <25%, 25%–50%, and >50% corresponding to low, moderate, and high degrees of heterogeneity, respectively. Then, we examined publication bias with the funnel plot and Egger’s test. All analyses were conducted in the statistical software Review Manager (RevMan version 5.3; The Nordic Cochrane Center, Copenhagen, Denmark). P-value < 0.05 was considered to be statistically significant.

## Results

### Search results and study characteristics

**Figure 1:**
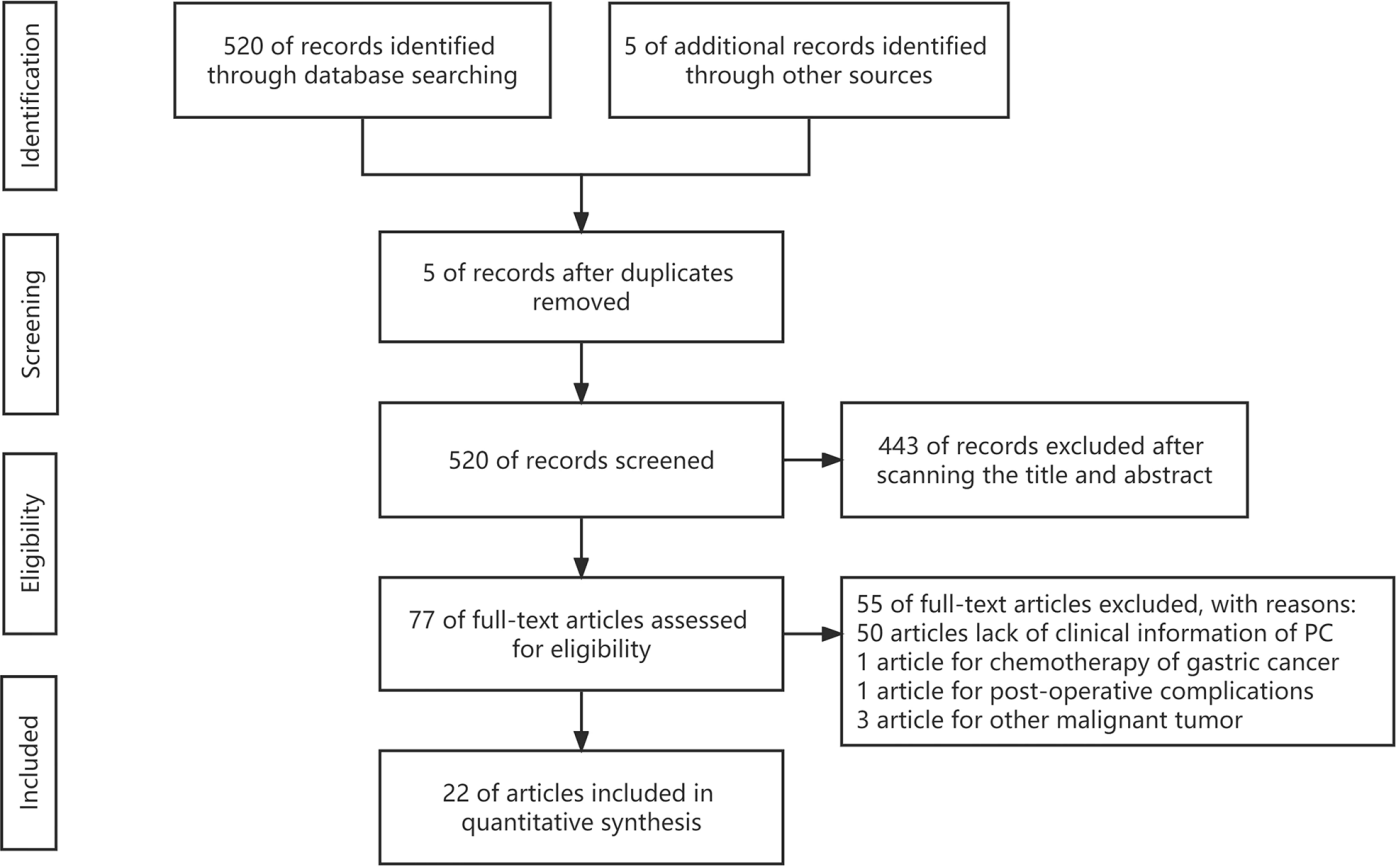
Flow diagram.

PRISMA flow chart is shown in **Figure 1**. There were 520 potentially relevant studies initially identified through the database according to the predefined search strategy. In addition, five studies were obtained manually. Five studies were excluded by duplicate checking. A total of 443 studies were excluded after scanning the title and abstract, and the remaining 77 studies were further reviewed by the full-text view. Of these 77 studies, 50 studies were removed because of the lack of clinical information of patients. Five studies were removed because the objects were not PC (one study about chemotherapy, one study about complications, and three studies focusing on other malignant tumors). Finally, 22 studies were included in the meta-analysis.

The characteristics of all studies are summarized in **Table 1**. The risk factors mentioned in the studies that may be related to PC and the number of studies for each factor are shown in **Table 2**. PC was defined as P1 and/or CY1 in nine studies, as only P1 in nine and only CY1 in four. The funnel charts of all results can be found in the **supplementary materials**.

**Table 1:** Baseline characteristics of the included studies.

Author and Year	Type of study	Duration of the study	Total patients	Positive cases	Definition of PC	Statistically significant risk factors in univariate analysis
Fujimura, 2002	Case-control study	1992.4–2000.4	39	21	P1 or CY1	CA125
Yano, 2000	Case-control study	1994.7–1998.5	32	13	P1 and CY uk	No factor
Hwang, 2004	Case-control study	199.7–2003.12	768	88	P1 and CY uk	CA125 CA199
Bentrem, 2005	Case-control study	1993.9–2002.12	371	24	CY1 and P0	No factor
Sarela, 2006	Case-control study	1993.4–2002.5	65	21	P1 and CY uk	N+ PD
Badgwell, 2008	Case-control study	1995.1–2005.12	381	39	CY1 and P0	No factor
La Torre, 2010	Case-control study	2003.7–2008.5	64	7	CY1 and P0	T4 N+ PD
Hur, 2010	Case-control study	2001.1–2005.12	589	72	P1 and CY uk	T4 N+ PD size
Kurita, 2010	Case-control study	2001.1–2008.3	236	41	P1 and CY uk	N2/3 PD Borrmann type IV size
Tsuchida, 2011	Case-control study	1999.6–2003.12	231	86	P1 or CY1	N+ Borrmann type IV
Strandby, 2015	Case-control study	2010–2012	219	21	P1 and CY uk	No factor
Lisiecki, 2015	Case-control study	2014.4–2015.7	51	12	CY1 and P uk	No factor
Ikoma, 2016	Case-control study	1995.1–2012.12	711	228	P1 or CY1	PD Borrmann type IV Signet-ring
Hu, 2016	Case-control study	2004.6–2014.5	582	138	P1 or CY1	T4 size Borrmann type IV
Li, 2017	Case-control study	2011.9–2013.9	249	39	P1 or CY1	Borrmann type IV
Hosogi, 2017	Case-control study	2006.5–2015.9	287	116	P1 or CY1	Borrmann type IV size LD
Huang, 2018	Case-control study	2008.12–2012.12	879	110	P1 or CY1	T4 N+ PD Borrmann type IV size
Rawicz Pruszyński, 2019	Case-control study	2016.8–2018.9	173	39	P1 and CY uk	T4 LD
Nakamura, 2019	Case-control study	2009.1–2017.12	35	28	P1 or CY1	No factor
Harris, 2019	Case-control study	2013.12–2016.10	363	75	P1 and CY uk	PD
Yang, 2020	Case-control study	2014.1–2019.4	672	89	P1 or CY1	T4 PD size
Zhao, 2020	cohort study	2015.4–2015.11	129	43	P1 and CY uk	PD CA125 LD CA199

uk, unknown; N+, positive lymph node metastasis; N2/3, N2 or N3 stage; PD, poorly differentiated carcinoma; Signet-ring, Signet-ring cell carcinoma; Size, tumor diameter ≥ 4 or 5 cm; LD, Lauren diffuse type.

**Table 2:** Risk factors and OR value of the pooled results.

Risk factor	OR value	Included studies	Sample size
T4	OR = 2.96, 95% CI: 1.87–4.69	11	3,877
N+	OR = 1.22, 95% CI: 0.86–1.73,	15	4,587
Poorly differentiated carcinoma	OR = 1.91, 95% CI: 1.42–2.56	14	4,424
Borrmann type IV	OR = 6.67, 95% CI: 3.33–13.36	8	2,924
Large tumor	OR = 5.12, 95% CI: 2.55–10.31	6	3,117
N2/3	OR = 2.38, 95% CI: 1.22–4.65	5	1,321
CA125 ≥ 37	OR = 19.45, 95% CI: 4.71–80.30	3	925
Lauren diffusion type	OR = 2.11, 95% CI: 1.60–2.79,	5	1,427
Signet-ring cell carcinoma	OR = 1.71, 95% CI: 1.30–2.26	4	1,328
CA199	OR = 4.22, 95% CI: 1.44–12.34	4	1,110
Gender	OR = 0.90, 95% CI: 0.74–1.09	9	4,054
Age	OR = 1.06,95% CI: 0.89–1.25	6	3,235

### The correlation between T stage and PC

Eleven studies, with 3,877 patients, from 2008 to 2020 were included (10, 14–23). The sample size of the studies varied from 49 to 879.

**Figure 2:**
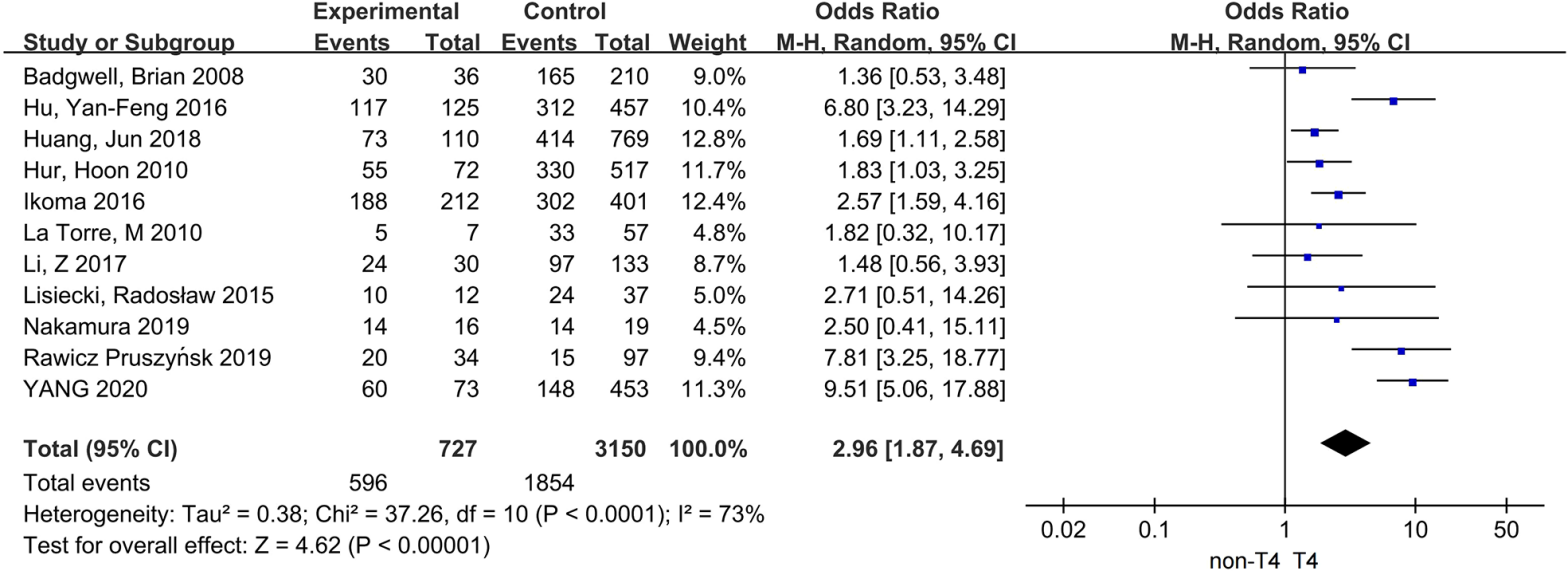
The correlation between T4 stage and PC.

The pooled results revealed that patients with T4 stage had a higher proportion in the PC group compared with the ones in the non-PC group; the difference was statistically significant; OR = 2.96, 95% CI: 1.87–4.69, P < 0.0001; I² = 73% (**Figure 2**).

### The correlation between N stage and PC

Two ways of classification were applied for the N stage. First, patients were divided into positive regional lymph node (N+) and negative (N−) groups, separately. In addition, 15 studies, with 4,587 patients, were included (10, 14–22, 24–28). Then, we divided patients into N0/1 and N2/3 groups, separately. According to this criteria, five studies, with 1,321 patients, were included (14, 17, 20, 22, 29). The sample size varied from 35 to 589.

**Figure 3:**
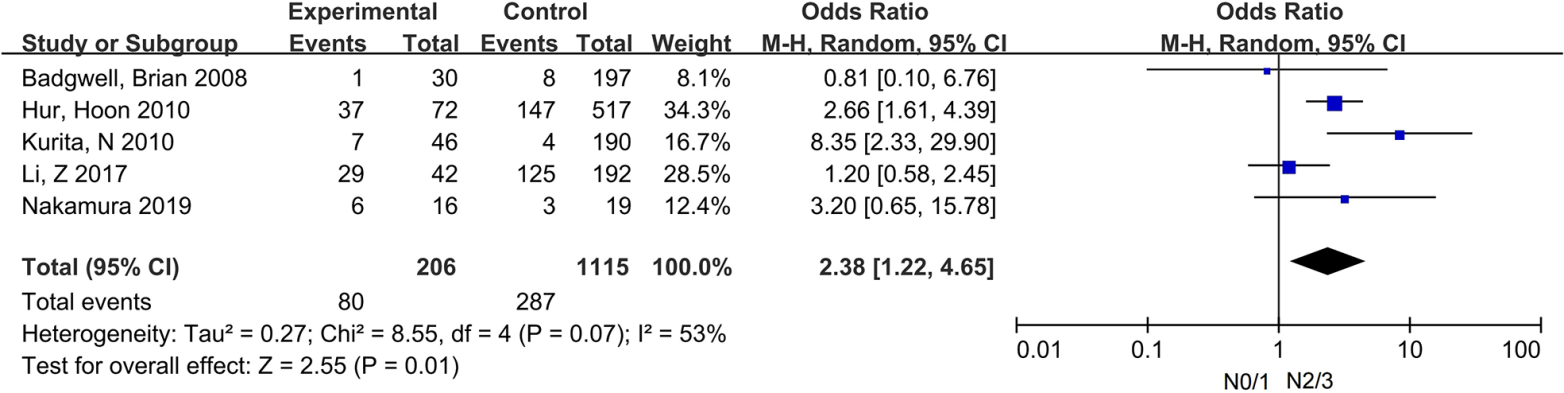
The correlation between N2/3 and PC.

There was no statistical correlation between N+ and PC, with OR = 1.22, 95% CI: 0.86–1.73, P < 0.0001; I² = 66%. On contrary, patients with N2/3 stage had a higher proportion in the PC group, with OR = 2.38, 95% CI: 1.22–4.65, P = 0.01; I² = 53% (**Figure 3**).

### The correlation between differentiation degrees and PC

Fourteen studies, with 4,424 patients, from 2006 to 2020 were included (15–23, 26, 27, 29–31). The sample size varied from 32 to 879.

**Figure 4:**
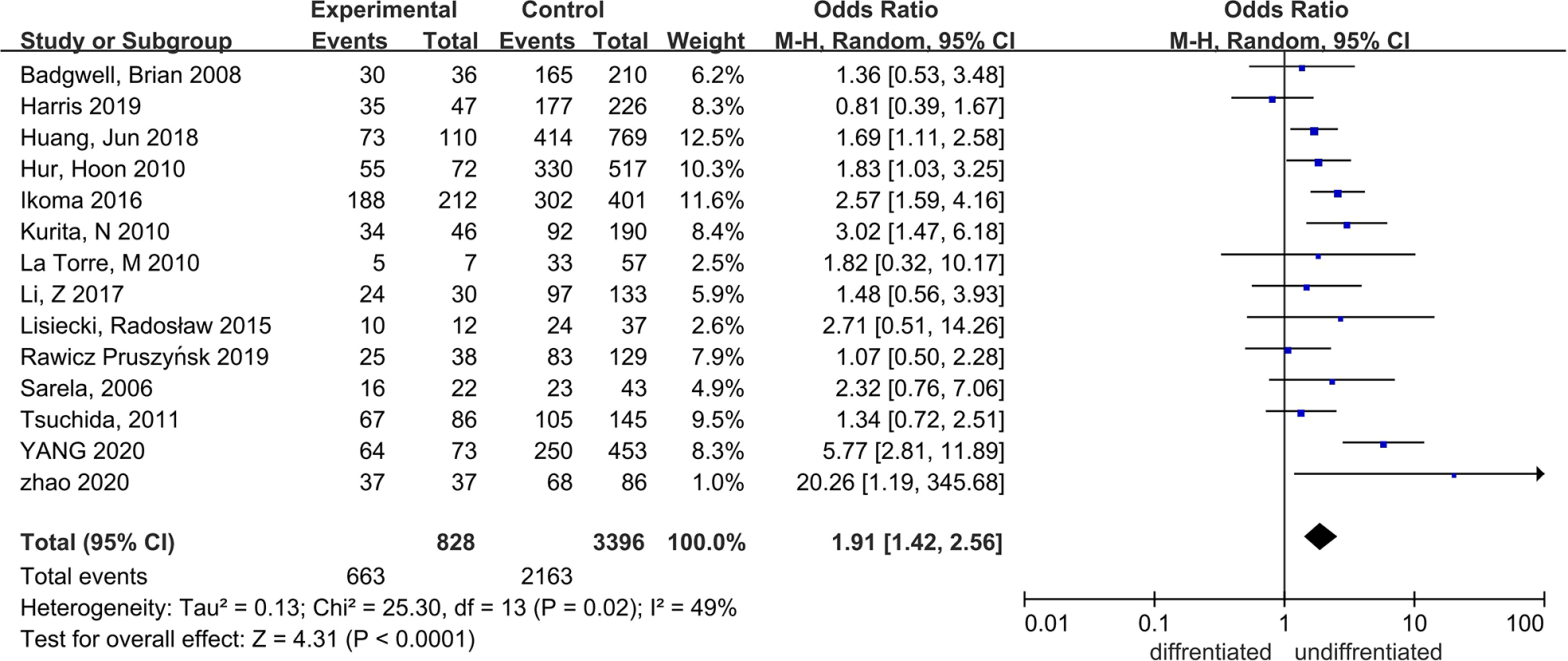
The correlation between differentiation degrees and PC.

The proportion of patients with poorly differentiated carcinoma was higher in the PC group, and the difference was statistically significant; OR = 1.91, 95% CI: 1.42–2.56, P < 0.0001; I² = 49% (**Figure 4**).

### The correlation between Borrmann type and PC

Eight studies, with 2,924 patients, from 2000 to 2018 were included (10, 16–18, 24, 26, 29, 32). The sample size of the studies varied from 32 to 879.

**Figure 5:**
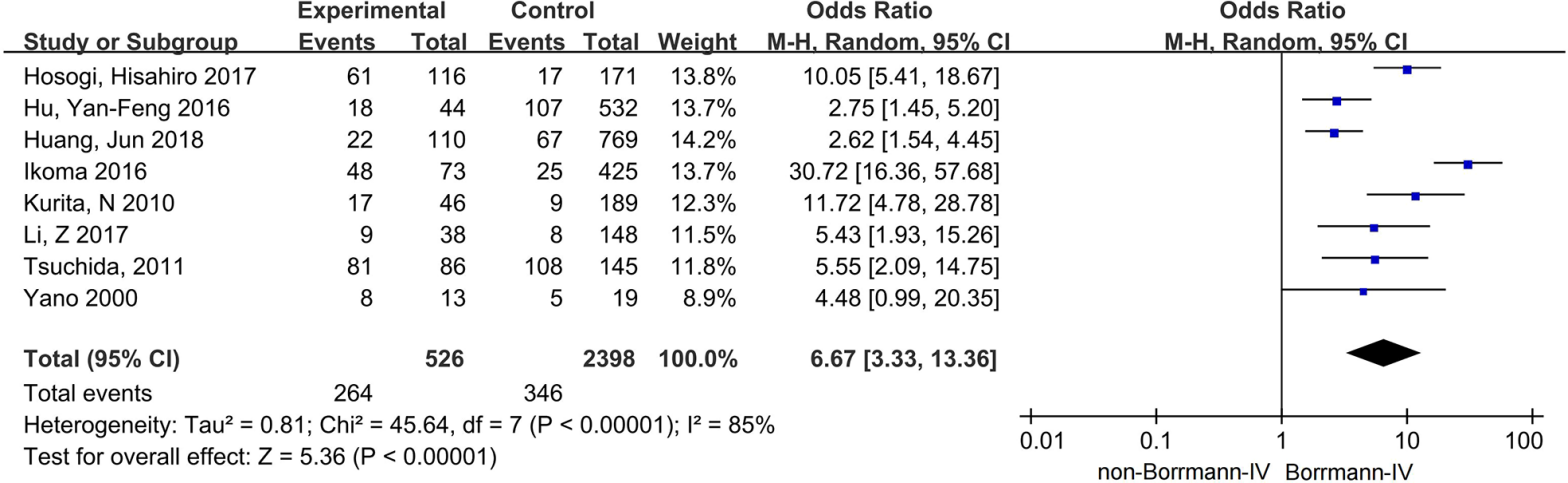
The correlation between Borrmann type IV and PC.

After summing up all the results, we could find that patients with Borrmann type IV had a higher proportion in the PC group, with OR = 6.67, 95% CI: 3.33–13.36, P < 0.0001; I² = 85% (**Figure 5**).

### The correlation between tumor diameter tumor and PC

Six studies recorded the diameters of the primary tumors, of which three studies used 4 cm as the cutoff value, and others used 5 cm. We put patients with tumor diameters ≥4 or ≥5 cm together as the large tumor size group. Finally, the number of the included patients was 3,117 (10, 16, 20, 23, 24, 29), with the sample size changing from 231 to 879.

**Figure 6:**
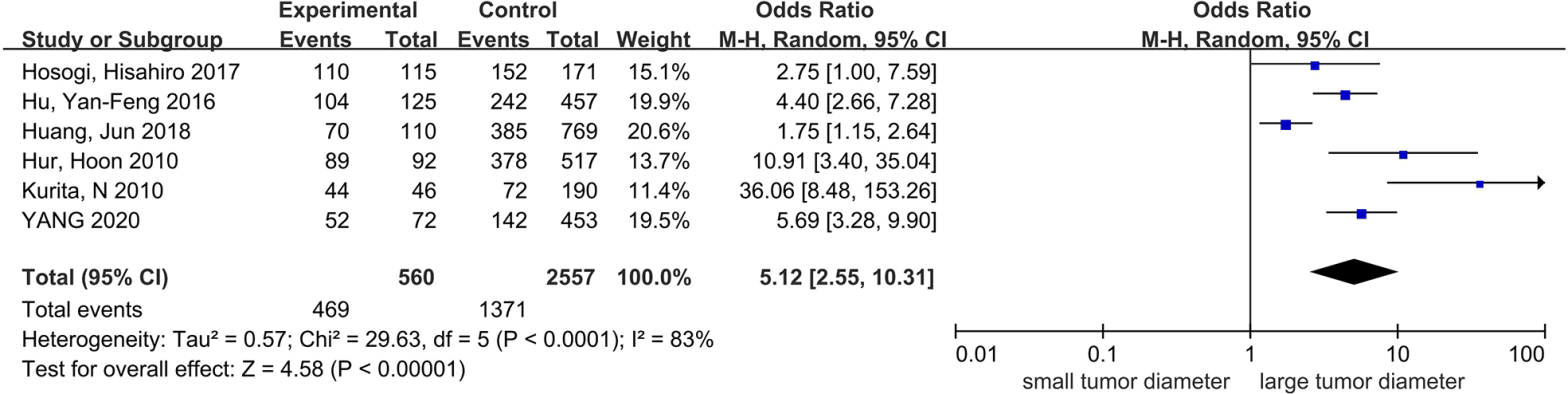
The correlation between tumor diameter and PC.

The pooled results showed that patients with large tumor size had a higher proportion in the PC group; the difference was statistically significant; OR = 5.12, 95% CI: 2.55–10.31, P < 0.0001; I² = 83% (**Figure 6**).

### The correlation between serum CA125 and PC

Three studies, with 925 patients, from 2002 to 2020 were included (11, 30, 33). All of the studies used 35 ng/ml as cutoff value. The sample size varied from 37 to 728.

**Figure 7:**
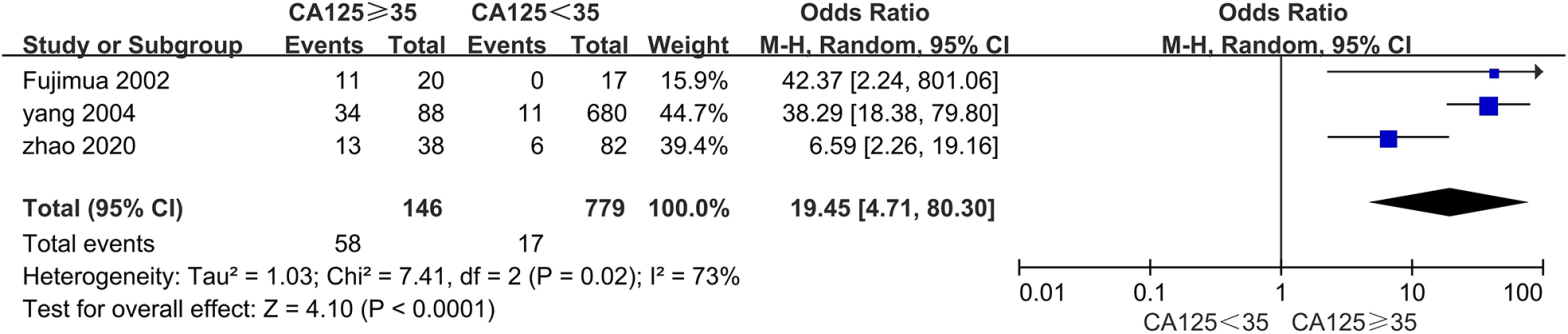
The correlation between serum CA125 and PC.

The pooled results showed that patients with serum CA125 ≥ 35 ng/ml had a higher proportion in the PC group, with OR = 19.45, 95% CI: 4.71–80.30, P < 0.0001; I² = 73% (**Figure 7**).

### The correlation between Lauren diffusion type and PC

Five studies, with 1,427 patients, from 2015 to 2020 were included (15, 16, 19, 24, 30). The sample size of the studies changed from 51 to 804.

The proportion of patients with Lauren diffusion type was higher in the PC group, with OR = 2.11, 95% CI: 1.60–2.79, P < 0.0001; I² = 71% (**Supplementary Figure 8**).

### The correlation between Signet-ring cell carcinoma and PC

Four studies, with 1,328 patients, from 2008 to 2017 were included (17, 18, 22, 25). The sample size changed from 185 to 662.

The pooled results showed that patients with Signet-ring cell carcinoma had a higher proportion in the PC group, with OR = 1.71, 95% CI: 1.30–2.26, P = 0.0001; I² = 0% (**Supplementary Figure 9**).

### The correlation between serum CA199 and PC

Four studies, with 1,110 patients, from 2002 to 2020, which used 37 ng/ml as cutoff value, were included (11, 17, 30, 33). The sample size of the studies varied from 37 to 768.

The pooled results showed that patients with the serum CA199 ≥ 37 ng/ml had a higher proportion in the PC group, with OR = 4.22, 95% CI: 1.44–12.34, P = 0.001; I² = 81% (**Supplementary Figure 10**).

### The correlation between gender and PC

Nine studies, with 4,054 patients, from 2005 to 2019 were included (10, 15–18, 20, 26, 28, 31). The sample size varied from 173 to 889.

The pooled results showed that the gender of patients was not statistically associated with PC, OR = 1.06, 95% CI: 0.89–1.25, P = 0.51; I² = 63% (**Supplementary Figure 11**).

### The correlation between age and PC

Five studies used 65 years old as the cutoff value and one for 60 years old. We put patients elder than 60 or 65 together as one group. Finally, six studies, with 3,235 patients, from 2005 to 2019 were included (10, 16, 18, 20, 28). The sample size varied from 153 to 878.

The pooled results showed that the age of patients was not statistically associated with PC, OR = 0.90, 95% CI: 0.74–1.09, P = 0.26; I² = 0% (**Supplementary Figure 12**).

## Discussion

The guidelines provide different indications of SL for patients with advanced gastric cancer. The NCCN (National Comprehensive Cancer Network) guideline recommends that all locoregional patients undergo SL with cytology (8). However, the JGCA (Japan Gastric Cancer Association) guideline recommends that only patients with large Borrmann type III or IV or bulky lymph nodes require SL (7). In between, the CSCO (Chinese Society of Clinical Oncology) guideline sets the indication as CT suspected PC or T3−4/N+ patients ready for neoadjuvant therapy (9). The conflict between the guidelines raised the need for further research on the risk factors of PC to select patients suitable for SL.

In this meta-analysis, we found that there was a cognitive gap between awareness and importance of risk factors of PC, which could partly explain the reasons for the current divergence. In addition to the clinical stage mentioned by the guidelines, there were also factors significantly statistically related to PC but with low concern, such as tumor size and CA125.

T stage is one of the most discussed factors, and our data support its relevance with PC. The majority of the included studies used T4 as a criterion, and T4’s relevance with PC is also consistent with the “seed and soil” hypothesis, which states that more free cancer cells exfoliated from the tumor penetrating serosa could lead to PC (34). However, if all three studies with CY1 only were included in the analysis, then OR (95% CI) was 1.65 (0.79–3.44), which means that there is no statistical difference (19, 21, 22). Yoshida et al. found the occurrence of CY1 or P1 in five of the 1,509 patients with early gastric cancer, and pathological analysis revealed that the primary tumor invaded the submucosa and metastasized to the regional lymph nodes through the lymph vessels; they speculated that tumor cells may metastasize to the peritoneal cavity through the lymphatic system rather than by breaking through the plasma (35). This could be the reason for this subgroup analysis. The analysis of the remaining eight studies involving P1 showed a stronger statistical correlation between T4 and P1. We prefer to recommend SL to patients with T4 stage to avoid missing PC, although the relationship between T4 and CY1 still needs to be explored.

N stage is another most mentioned factor in the studies and guidelines, but the statistical relevance is not obvious. An alternative approach is for N0/1 and N2/3 groups. Although with fewer studies and increased publication bias, there is a significant increase in OR (95% CI) of 2.38 (1.22–4.65). Yoshida et al. proposed that PC can occur through the lymphatic system (35). According to this hypothesis, the more regional lymph nodes that metastasize, the greater likelihood that tumor cells initiate PC by lymph vessels. We cannot propose a definitive SL strategy based on the N stage with insufficient evidence. It may be appropriate to analyze the relationship between the N1–3 stage and PC, separately.

Tumor size is often omitted in the guidelines, with only the Japanese guideline recommending SL for patients with Borrmann type III tumor with diameter ≥ 8 cm (7). However, multiple studies have confirmed the dependency between tumor diameter and PC. Although different lengths were used as the standard of classification, such as 4 or 5 cm, the results indicated that larger tumor diameter was independent risk factor of PC. However, there was no detailed description of how to measure tumor size in the studies. We suggest that patients with large tumor size undergo SL, but the method of measuring the diameter and the cutoff value of tumor size needs to be further clarified.

Several studies have concluded that Borrmann type IV is risk factor for PC, similarly to the JGCA guideline (10, 16–18, 24, 26, 29). In addition, the results of studies indicated that Borrmann type III is also risk factor for PC (10, 16, 26). This may be because Bormann type IV and large Borrmann type III tumors are usually accompanied by larger tumor size and more advanced stage. As discussed previously, patients with risk factors of PC, such as T4 and N+, are more likely to develop PC. Therefore, we recommend that patients with Bormann type IV and large Borrmann type III gastric cancer receive SL.

Although differentiation degrees have been widely studied, no definite conclusion has been made about its relationship with PC. The pooled results suggested that poorly differentiated carcinoma is a risk factor for PC, but the OR was only 1.91 and eight of the 14 studies had opposite results, with a weight of 45.2%. These diminish the confidence of the conclusion. Furthermore, the result of subgroup analysis showed that poorly differentiated carcinoma was not risk factor for CY1 but a risk factor for P1. This suggests that the relationship between poorly differentiated carcinoma and P1 and CY1 may be investigated, separately.

Regarding CA125, CA199, Signet-ring cell carcinoma, and Lauren’s diffuse type, the insufficient studies in the meta-analysis and heterogeneity between results reduce the confidence of the conclusion. We believe that only a system review of their relationship with PC can be made on the base of the current finding. In particular, the OR of elevated CA125 was 19.45, significantly higher than other factors. Moreover, all three studies indicated that elevated CA125 was a risk factor for PC (11, 30, 33). Tumor cells can cause CA125 elevation, and the mesothelial cells in the abdomen and pelvis stimulated by tumors can also increase CA125 secretion. Therefore, we suggest that patients with elevated CA125 should undergo SL and more attention should be paid to its relationship with PC.

There are still limitations in this study. The included studies used different definitions of PC. Four studies defined CY1 as PC and the remaining studies involved P1, of which the conclusions were generally consistent with the overall results. However, no similar relationship was observed in the subgroup analysis of CY1. For example, the relationship between T4 and CY1 was different from between T4 and PC or P1. Moreover, part of the conclusions was based on insufficient evidence, for instance, the relationship between CA125 and PC. In addition, there is heterogeneity and publication bias among the results of the included studies. Meanwhile, the absence of randomized controlled trials has led to the inclusion of only retrospective studies in this article.

In conclusion, this meta-analysis raised the potential conflict between current indications of SL and their actual relevance with PC. We think that patients with T4 stage, Borrmann type IV, large tumor size, and elevated CA125 are more likely to have PC and should undergo SL. In particular, the relationship between CA125 and PC deserves further investigation.

## Data availability statement

The original contributions presented in the study are included in the article/**Supplementary Material**. Further inquiries can be directed to the corresponding author.

## Author contributions

Conception and design of the research: ZYL, GG, and ZML. Analysis and interpretation of the data: GG, ZML, XY, and QW. Writing of the manuscript: GG and ZML. Critical revision of the manuscript for intellectual content: ZYL, GG, and ZML. Final review: ZYL, GG, ZML, and FS. All authors contributed to the article and approved the submitted version.

## Funding

This work was funded by Beijing Municipal Administration of Hospitals Incubating Program (PX2022047), Beijing Municipal Health Commission (DFL20181103 and ZYLX201701), and Clinical Medicine Plus X – Young Scholars Project, Peking University.

## Conflict of interest

The authors declare that the research was conducted in the absence of any commercial or financial relationships that could be construed as a potential conflict of interest.

## Publisher’s note

All claims expressed in this article are solely those of the authors and do not necessarily represent those of their affiliated organizations, or those of the publisher, the editors and the reviewers. Any product that may be evaluated in this article, or claim that may be made by its manufacturer, is not guaranteed or endorsed by the publisher.
