# Supplementary material for: Risk factors associated with peritoneal carcinomatosis of gastric cancer in staging laparoscopy: A systematic review and meta-analysis
Source: Front Oncol. 2022 Oct 28;12:955181. doi: 10.3389/fonc.2022.955181 (PMC9650136; doi:10.3389/fonc.2022.955181)
Supplement: Supplementary file 1 [file DataSheet_1.pdf]

Study or Subgroup	not diffused type Events	Total	lauren diffused type Events	Total	Weight	Odds Ratio M-H, Fixed, 95% CI	Odds Ratio M-H, Fixed, 95% CI
Hosogi, Hisahiro 2017	100	116	98	171	15.5%	4.66 [2.53, 8.56]	
Huang, Jun 2018	61	106	360	698	57.1%	1.27 [0.84, 1.92]	
Lisiecki, Radosław 2015	9	12	14	39	2.3%	5.36 [1.24, 23.10]	
Rawicz Pruszyńsk 2019	25	39	57	125	13.8%	2.13 [1.01, 4.48]	
zhao 2020	18	39	23	82	11.3%	2.20 [1.00, 4.86]	
**Total (95% CI)**		**312**		**1115**	**100.0%**	**2.11 [1.60, 2.79]**	
Total events	213		552				

Heterogeneity: Chi² = 13.84, df = 4 (P = 0.008); I² = 71%
Test for overall effect: Z = 5.29 (P < 0.00001)

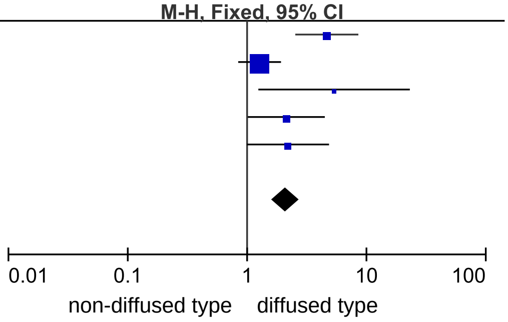

Fig.8 The correlation between lauren diffused type and PC

Study or Subgroup	Experimental Events	Experimental Total	Control Events	Control Total	Weight	Odds Ratio M-H, Random, 95% CI
Badgwell, Brian 2008	20	39	102	223	16.2%	1.25 [0.63, 2.47]
Ikoma 2016	131	321	97	341	71.8%	1.73 [1.25, 2.40]
Li, Z 2017	6	37	12	148	6.8%	2.19 [0.76, 6.30]
Strandby, R 2015	4	21	15	198	5.1%	2.87 [0.86, 9.62]
**Total (95% CI)**		**418**		**910**	**100.0%**	**1.71 [1.30, 2.26]**
Total events	161		226			

Heterogeneity: Tau² = 0.00; Chi² = 1.75, df = 3 (P = 0.63); I² = 0%

Test for overall effect: Z = 3.85 (P = 0.0001)

Odds Ratio
M-H, Random, 95% CI

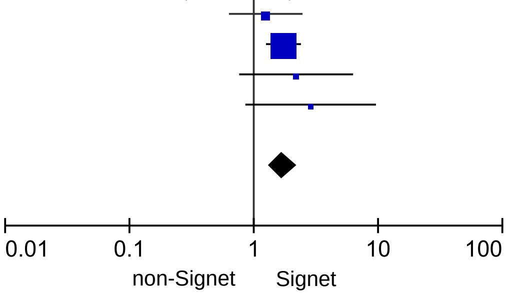

Fig.9 The correlation between Signet-ring cell carcinoma and PC

Study or Subgroup	Experimental Events	Experimental Total	Control Events	Control Total	Weight	Odds Ratio M-H, Random, 95% CI
Fujimura, T 2002	6	20	5	17	20.5%	1.03 [0.25, 4.24]
Li, Z 2017	8	36	16	143	25.6%	2.27 [0.88, 5.82]
yang 2004	33	88	34	680	29.4%	11.40 [6.56, 19.81]
zhao 2020	15	40	6	86	24.5%	8.00 [2.81, 22.81]
**Total (95% CI)**		**184**		**926**	**100.0%**	**4.22 [1.44, 12.34]**
Total events	62		61			

Heterogeneity: $Tau^2 = 0.94$; $Chi^2 = 15.85$, df = 3 (P = 0.001); $I^2 = 81\%$

Test for overall effect: Z = 2.63 (P = 0.009)

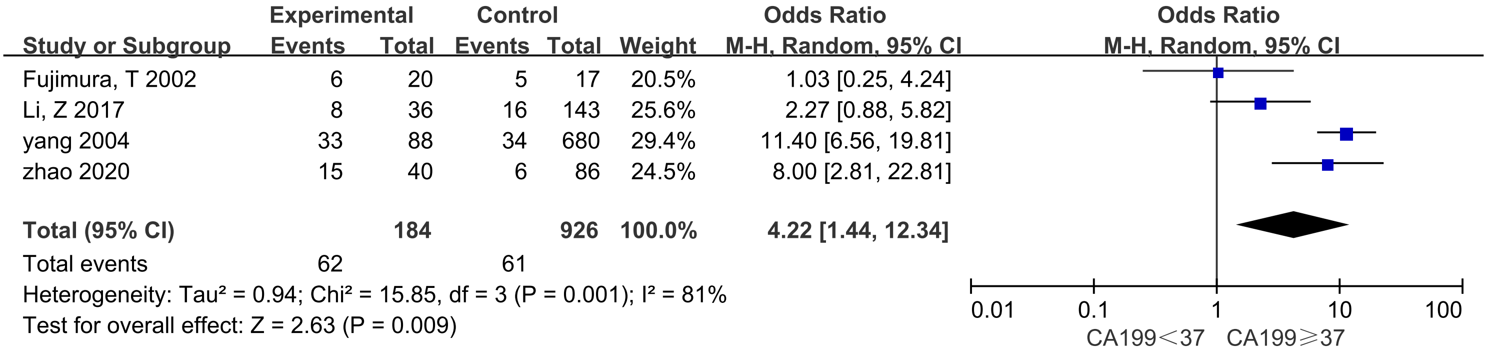

Fig.10 The correlation between serum CA199 and PC

Study or Subgroup	Experimental Events	Experimental Total	Control Events	Control Total	Weight	Odds Ratio M-H, Fixed, 95% CI
Bentrem 2005	16	24	246	347	4.0%	0.82 [0.34, 1.98]
Harris 2019	47	75	204	288	11.9%	0.69 [0.41, 1.18]
Hu, Yan-Feng 2016	328	457	69	125	11.5%	2.06 [1.37, 3.10]
Huang, Jun 2018	67	110	525	779	19.2%	0.75 [0.50, 1.14]
Hur, Hoon 2010	54	72	364	514	8.4%	1.24 [0.70, 2.18]
Ikoma 2016	144	228	290	434	27.8%	0.85 [0.61, 1.19]
Li, Z 2017	30	38	105	149	3.4%	1.57 [0.67, 3.70]
Rawicz Pruszyńsk 2019	23	39	90	134	6.3%	0.70 [0.34, 1.46]
Tsuchida, 2011	59	86	89	155	7.5%	1.62 [0.93, 2.82]
**Total (95% CI)**		**1129**		**2925**	**100.0%**	**1.06 [0.89, 1.25]**
Total events	768		1982			

Heterogeneity: $Chi^2 = 21.91$, df = 8 (P = 0.005); $I^2 = 63\%$
Test for overall effect: Z = 0.65 (P = 0.51)

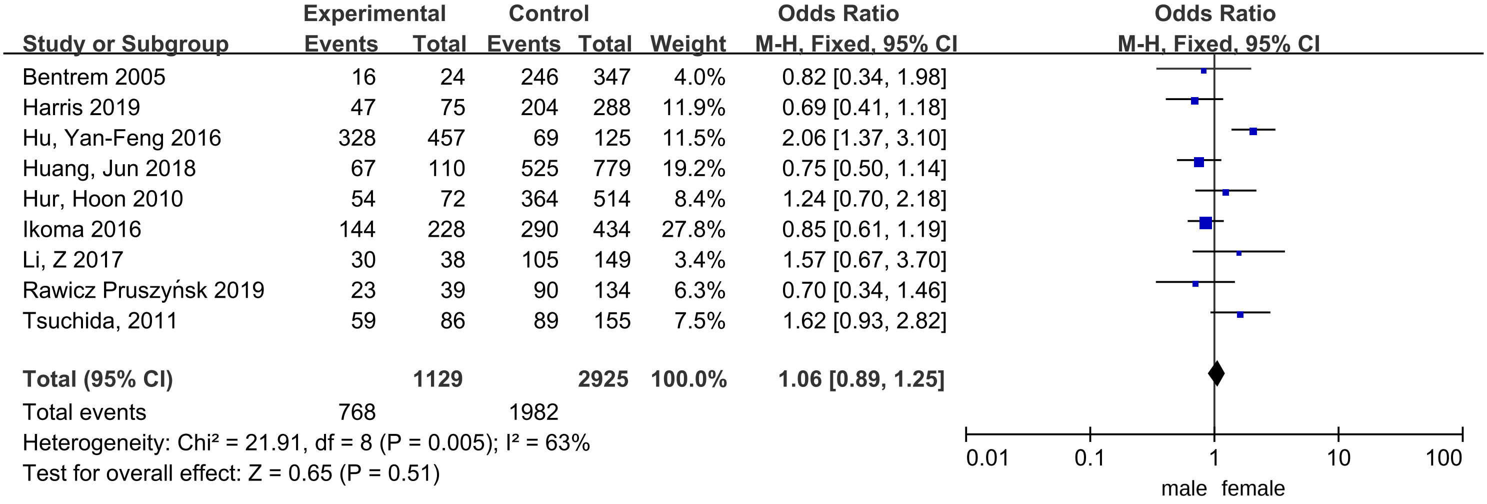

Fig.11 The correlation between gender and PC

Study or Subgroup	Experimental Events	Experimental Total	Control Events	Control Total	Weight	Odds Ratio M-H, Fixed, 95% CI	Odds Ratio M-H, Fixed, 95% CI
Bentrem 2005	14	24	162	347	4.0%	1.60 [0.69, 3.70]	
Hu, Yan-Feng 2016	123	457	37	125	19.3%	0.88 [0.57, 1.35]	
Huang, Jun 2018	47	109	371	769	23.8%	0.81 [0.54, 1.22]	
Hur, Hoon 2010	19	72	163	517	13.3%	0.78 [0.45, 1.36]	
Ikoma 2016	78	228	165	434	34.0%	0.85 [0.61, 1.19]	
Rawicz Pruszyńsk 2019	22	39	55	114	5.6%	1.39 [0.67, 2.89]	
**Total (95% CI)**		**929**		**2306**	**100.0%**	**0.90 [0.74, 1.09]**	
Total events	303		953				

Heterogeneity: Chi² = 3.79, df = 5 (P = 0.58); I² = 0%

Test for overall effect: Z = 1.12 (P = 0.26)

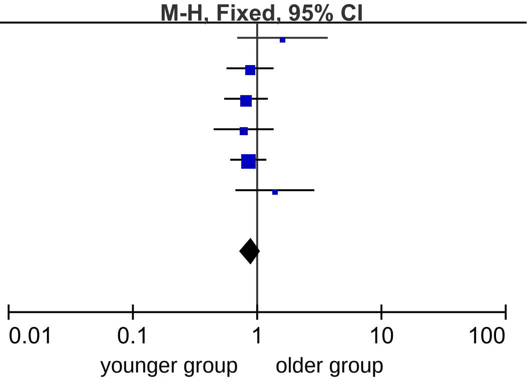

Fig.12 The correlation between age and PC
