# Supplementary material for: Risk factors associated with peritoneal carcinomatosis of gastric cancer in staging laparoscopy: A systematic review and meta-analysis
Source: Front Oncol. 2022 Oct 28;12:955181. doi: 10.3389/fonc.2022.955181 (PMC9650136; doi:10.3389/fonc.2022.955181)
Supplement: Supplementary file 2 [file DataSheet_2.pdf]

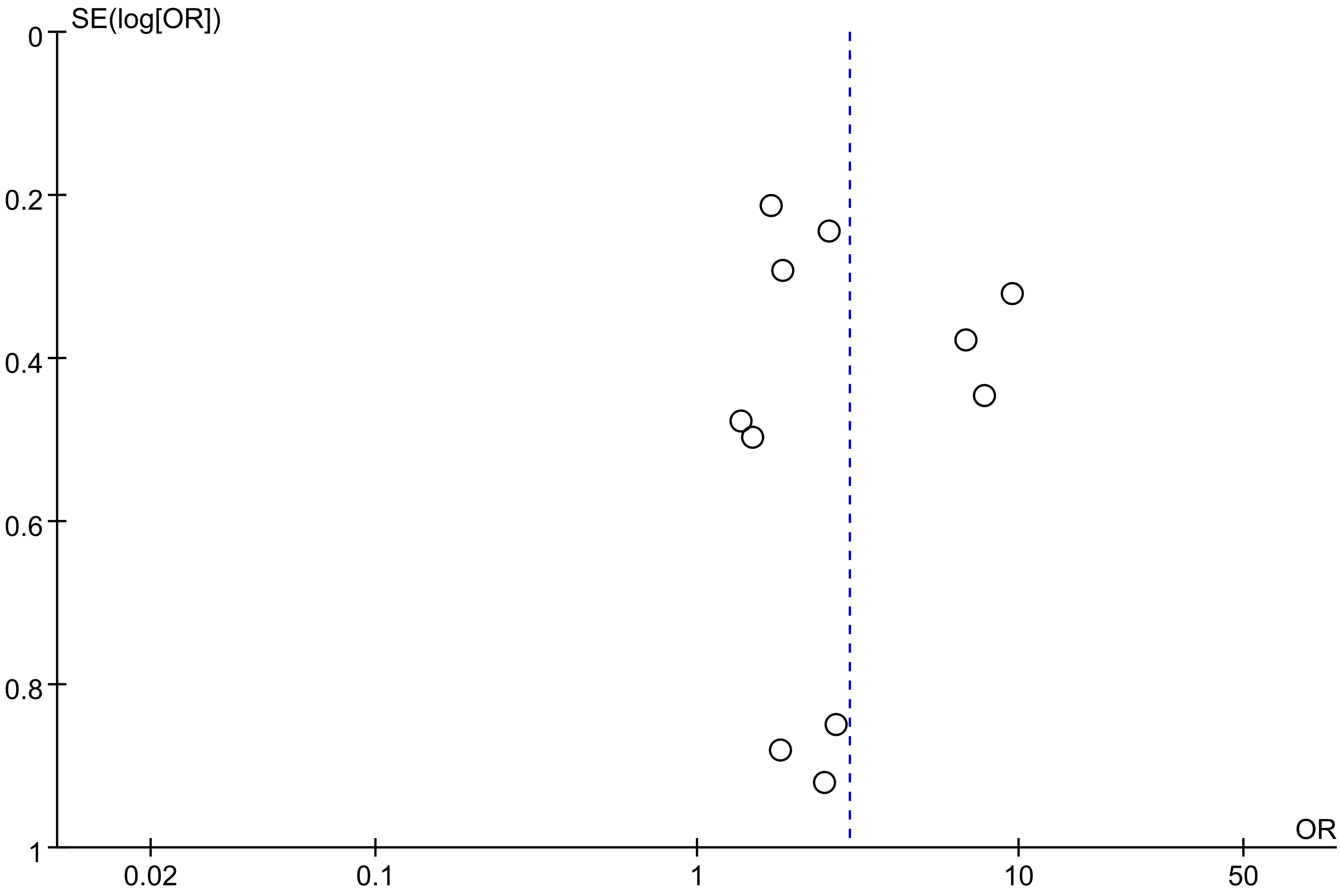

Fig.13 Funnel plot of T4 stage

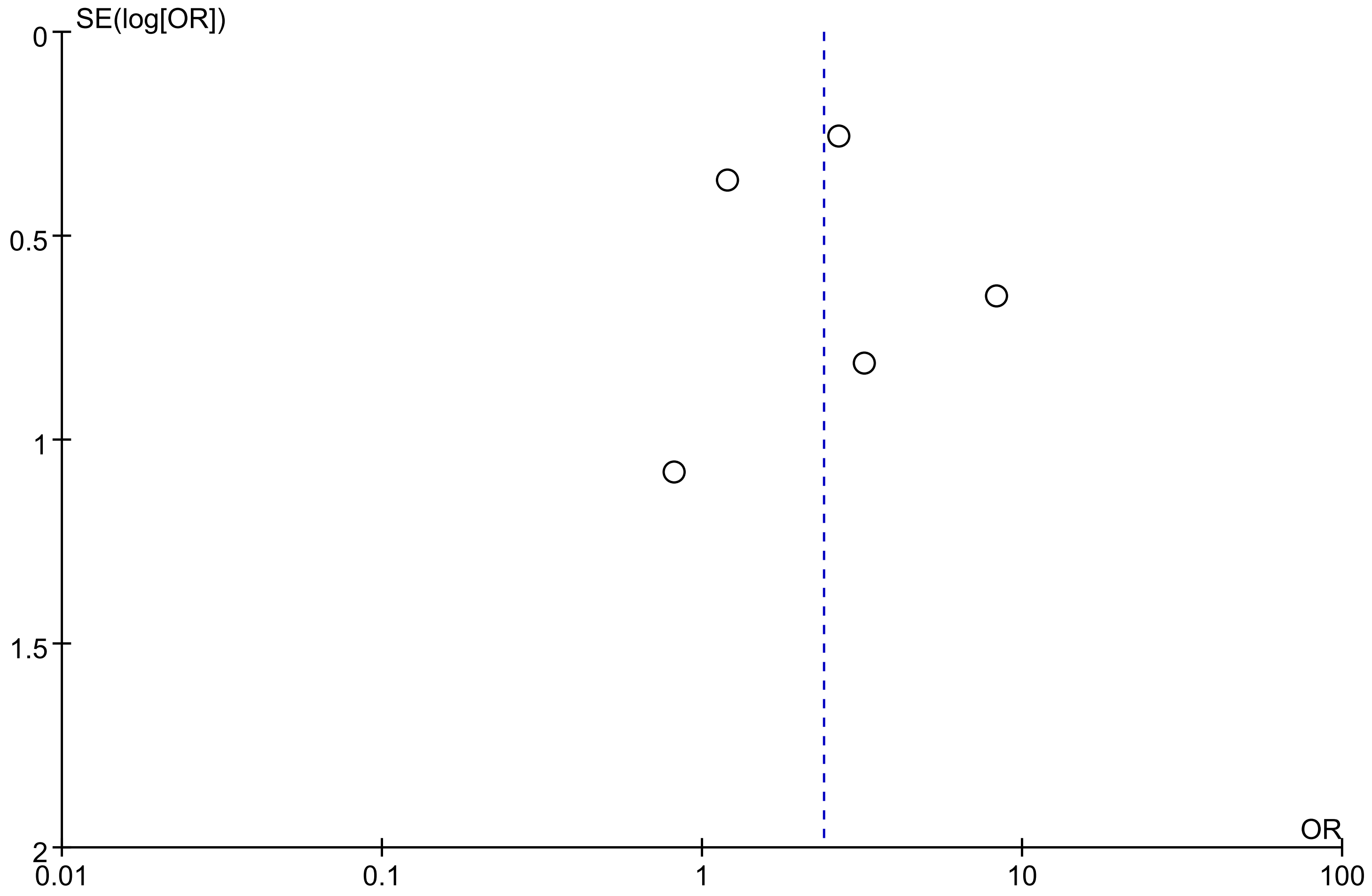

Fig.14 Funnel plot of N2/3 stage

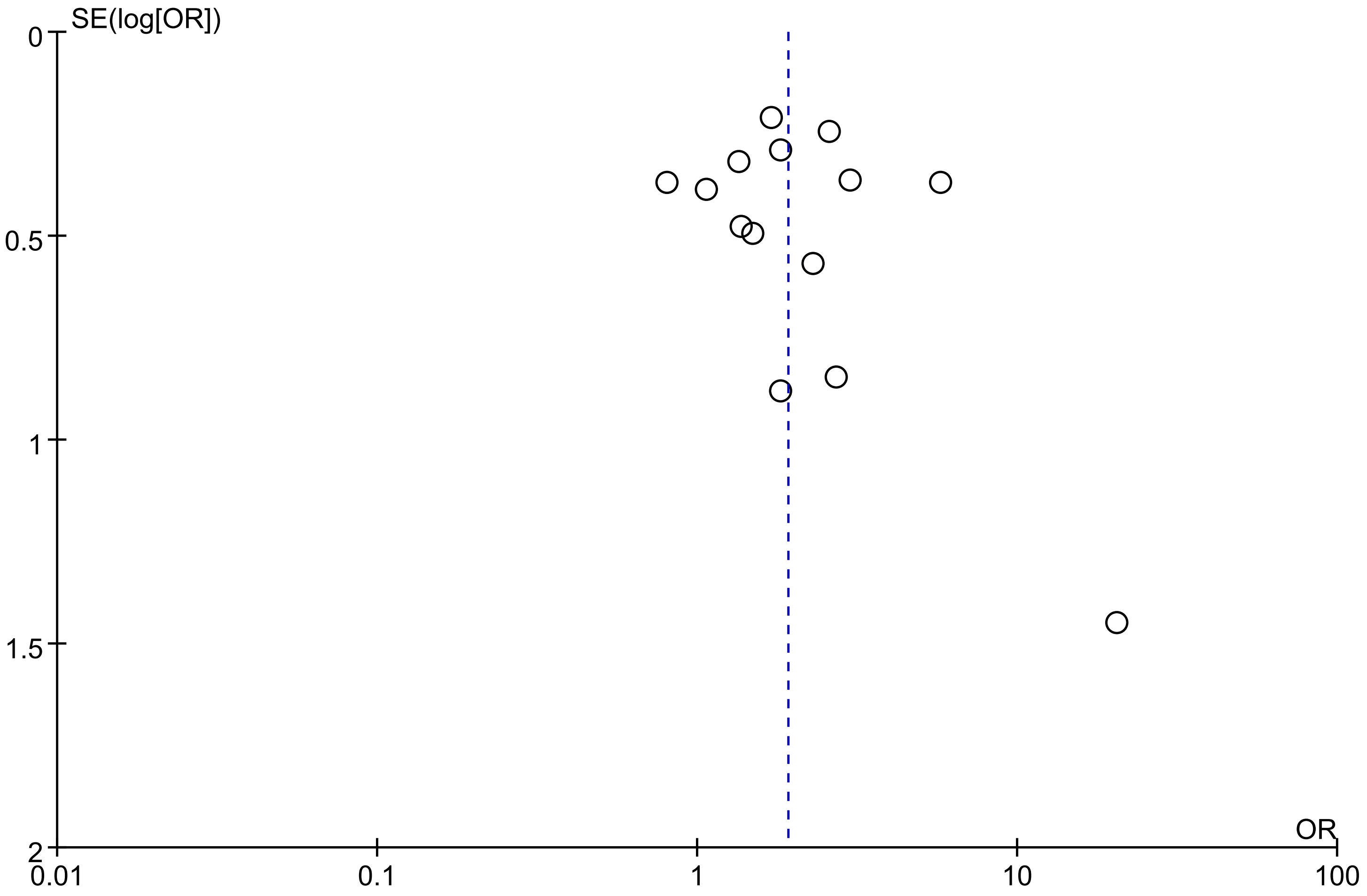

Fig.15 Funnel plot of undifferentiated carcinoma

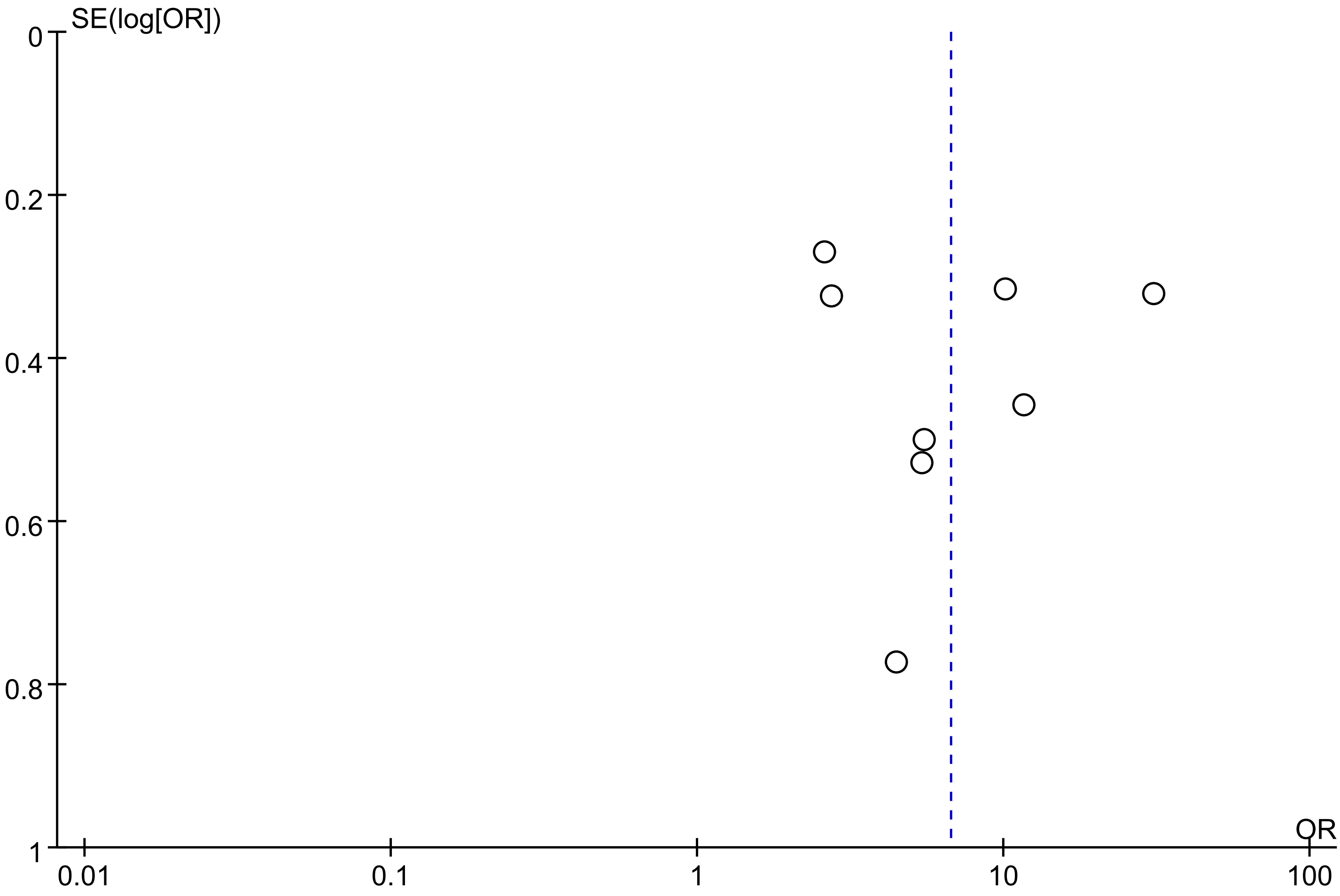

Fig.16 Funnel plot of Borrmann-IV type

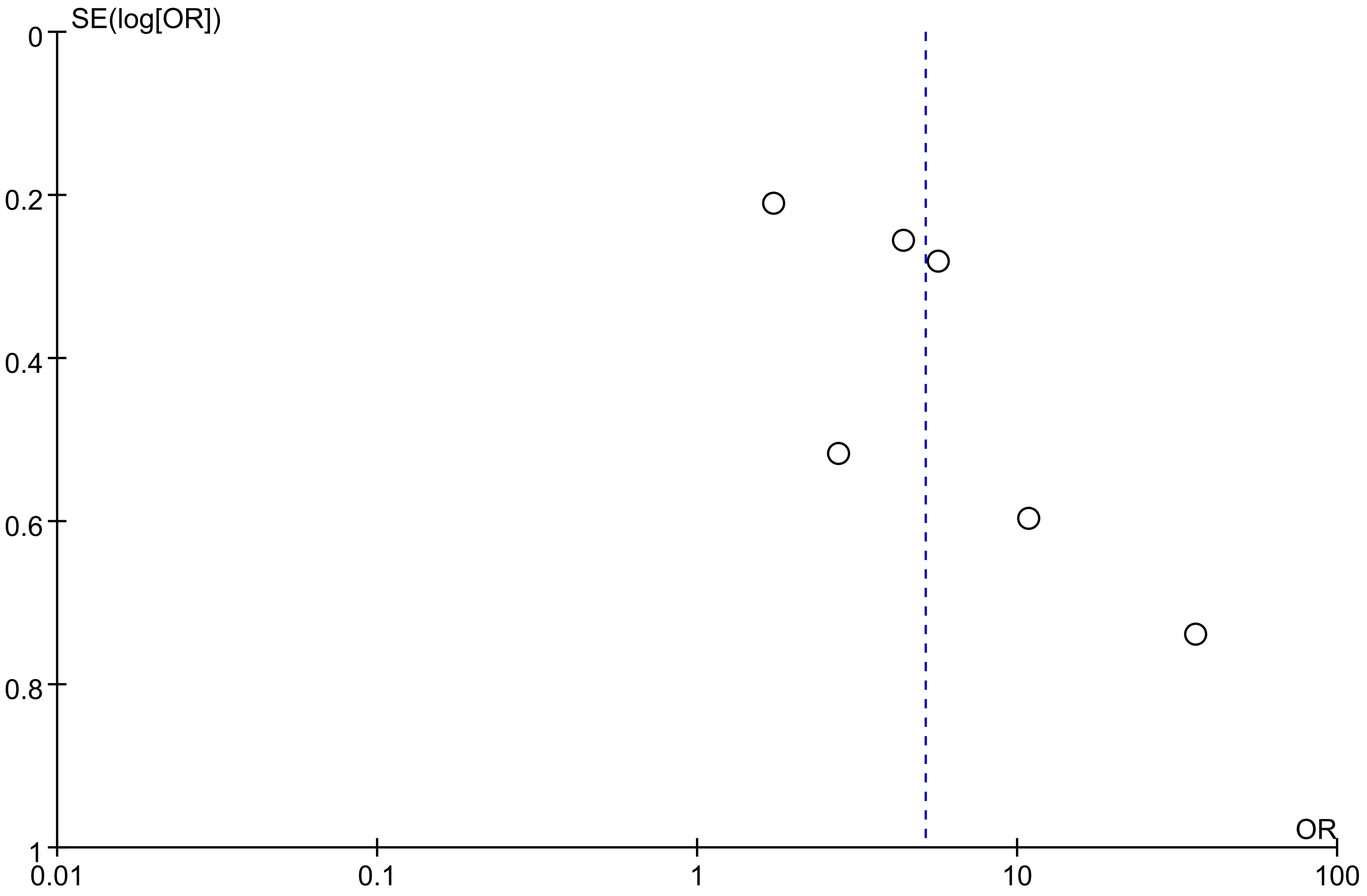

Fig.17 Funnel plot of large tumor diameter

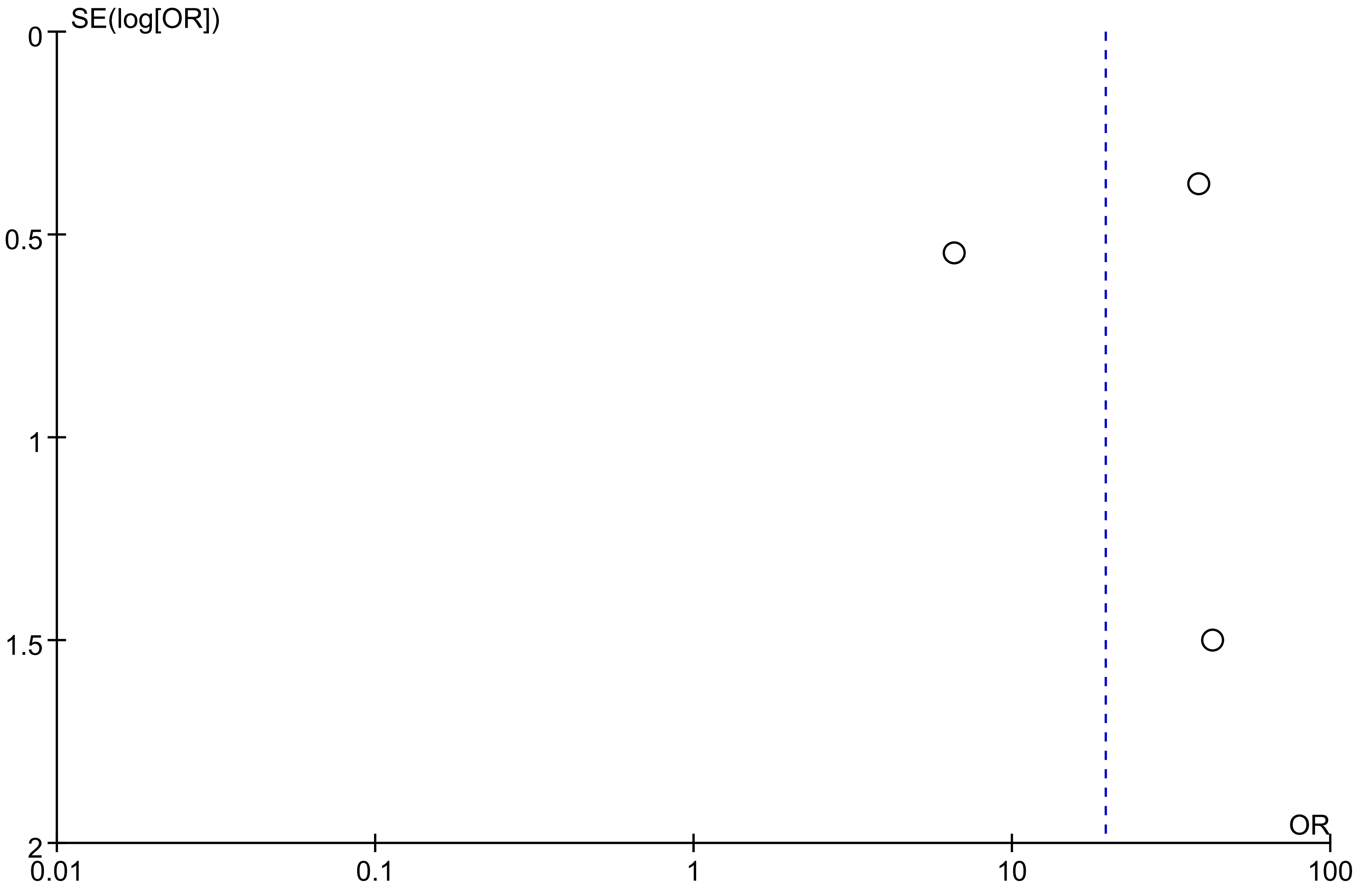

Fig.18 Funnel plot of serum CA125

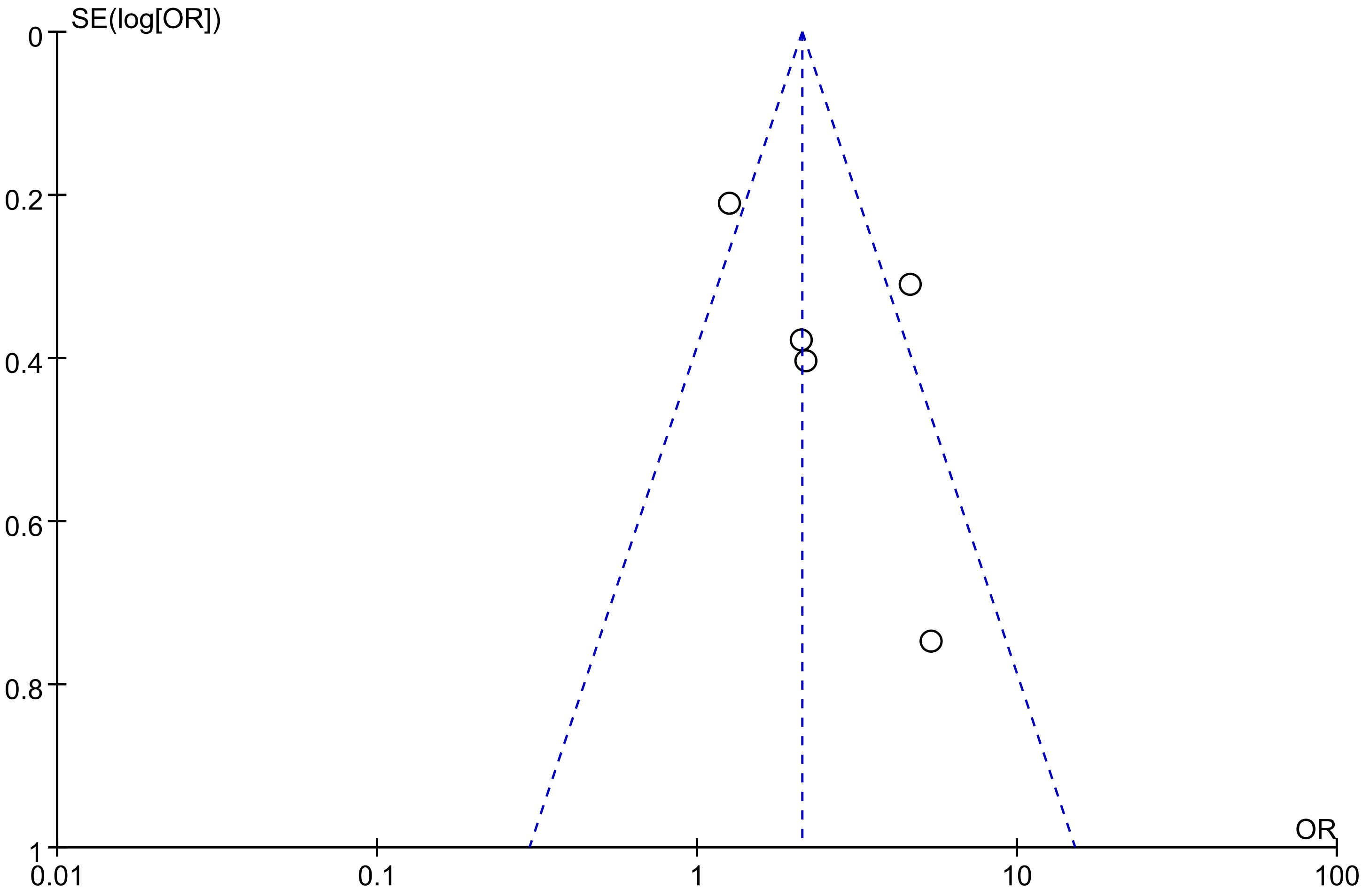

Fig.19 Funnel plot of lauren diffused type

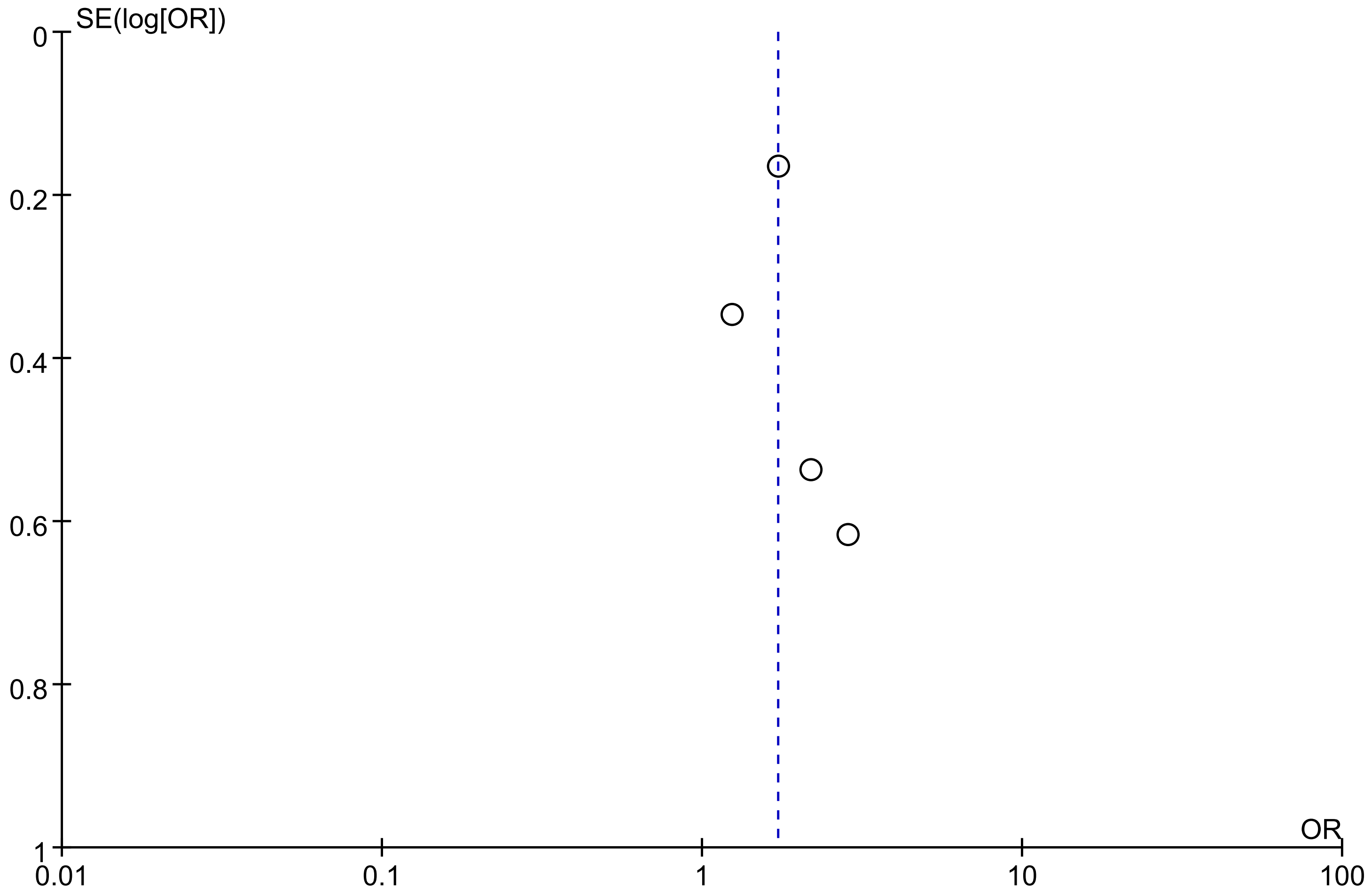

Fig.20 Funnel plot of Signet-ring cell carcinoma

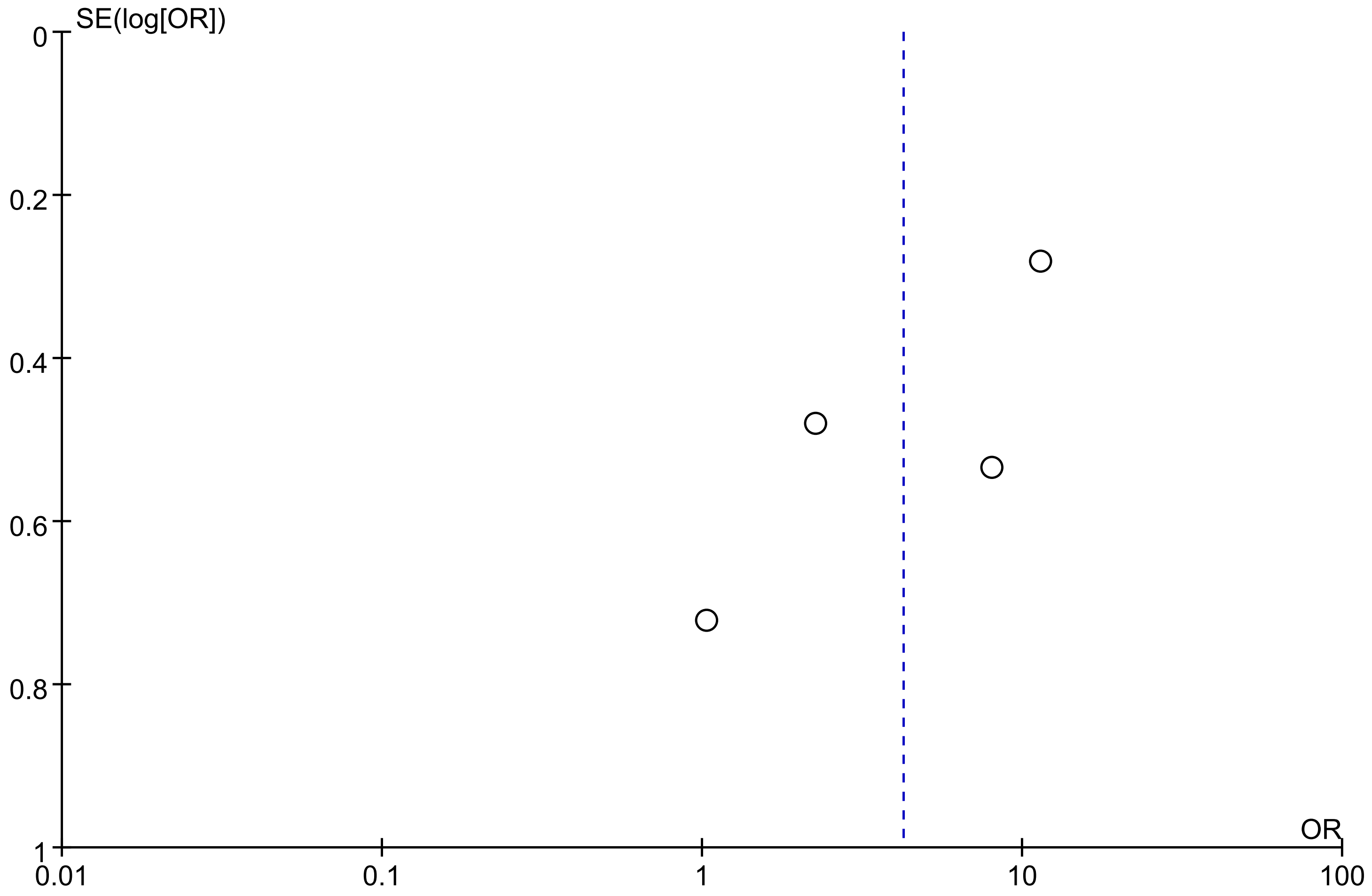

Fig.21 Funnel plot of serum CA199

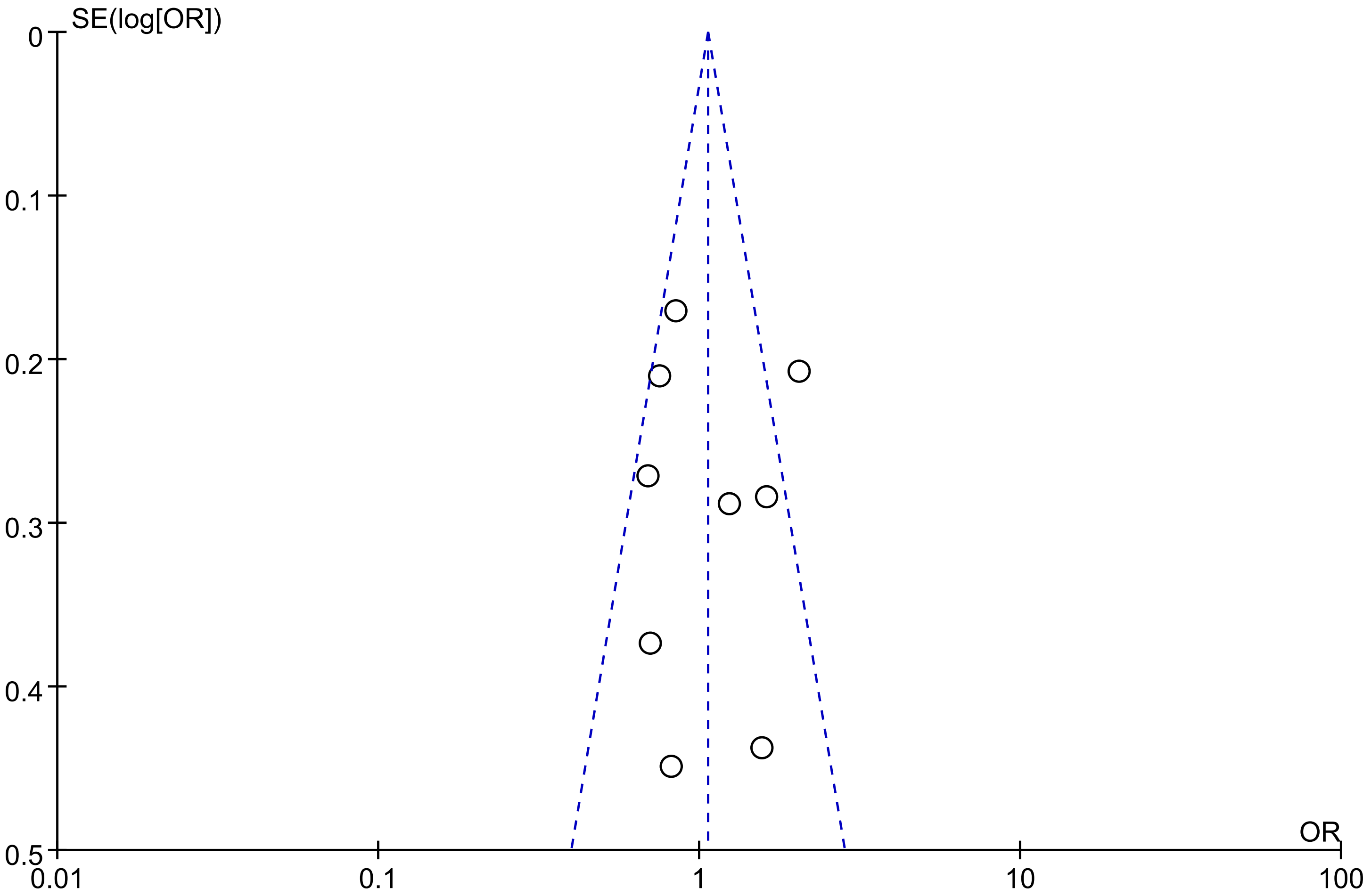

Fig.22 Funnel plot of gender

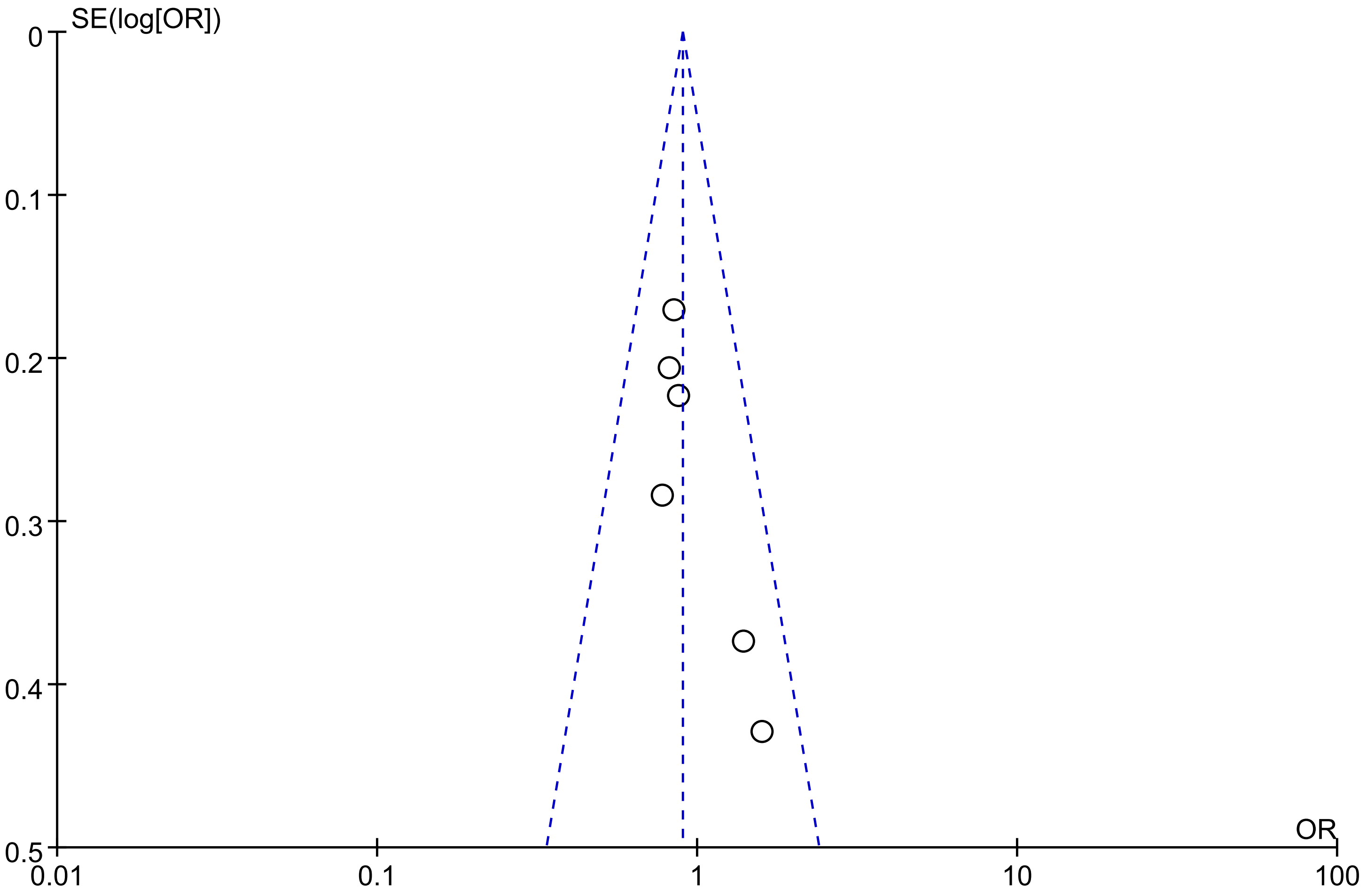

Fig.23 Funnel plot of age
